# Swelling, Serosal Adhesion, Protein Adsorption, and Biocompatibility of Pectin–TEOS Gels

**DOI:** 10.3390/gels11120984

**Published:** 2025-12-07

**Authors:** Nikita Paderin, Alisa Sokolova, Sergey Popov

**Affiliations:** Institute of Physiology of Federal Research Centre “Komi Science Centre of the Urals Branch of the Russian Academy of Sciences”, 50 Pervomaiskaya Str., 167982 Syktyvkar, Russia; paderin_nm@mail.ru (N.P.); alisasokolova.ru@yandex.ru (A.S.)

**Keywords:** pectin, hydrogel, swelling, serosal adhesion, biocompatibility, protein adsorption, leukocyte adhesion

## Abstract

The objective of this study was to develop a pectin–tetraethoxysilane (TEOS) hybrid gel with improved functional properties and biocompatibility. The sol–gel process was used to create pectin–TEOS hydrogels containing 0.75, 1.00, 1.25, and 1.50 M TEOS, which were labeled AP-T0.75, AP-T1.00, AP-T1.25, and AP-T1.50. The pectin–TEOS hydrogel AP-T1.50 exhibited a hardness of 631 kPa, a Young’s modulus of 1588 kPa, and an elasticity of 1.95 mm. The degree of swelling decreased as the TEOS content increased. The pectin–TEOS hydrogel AP-T1.25 exhibited the highest strength of adhesion to serosa of 60.6 mN. Serum protein and bovine serum albumin (BSA) adsorption by pectin–TEOS gels was recorded in the range of 2–43 µg/mg after 6 h of incubation at pH 5.0, 7.4, and 8.0. Pectin–TEOS gels demonstrated low rates of hemolysis and complement activation. Leukocyte adhesion on the surface of pectin–TEOS gels depends on TEOS content. Consequently, the mechanical characteristics, serosal adherence, and biocompatibility of pectin–TEOS gel position it as a strong contender for the advancement of smart biomaterials.

## 1. Introduction

Plant-derived polysaccharides are considered promising candidates for the development of biocompatible gel materials [1,2]. Among other plant polysaccharides, pectin-based gel materials have been investigated in wound dressing [3,4], drug delivery [5], and other biomedical fields [6]. It is recognized that pectins form the gel matrix of the plant wall of angiosperms and are distinguished by their structural diversity due to the heterogeneity of the pectin macromolecule’s structure [1]. Their softness, wetness, responsiveness, and biocompatibility contribute to intensive research on pectin hydrogels as biomaterials for tissue engineering [7]. To obtain gel biomaterials, commercial apple or citrus pectins are commonly used, which are predominantly composed of a linear chain of 1,4-α-D-galacturonic acid residues. However, despite promising results, pectin-based tissue engineering biomaterials are hindered by several limitations, such as low mechanical strength and stability in physiological environments, in addition to the ability to interact with endogenous proteins, which carries the risk of implant rejection. Pectin has been found to adhere to the glycocalyx of the visceral mesothelium [8]; however, it is noted that the tissue adhesion of pectin hydrogels should be enhanced to better predict the localization of biomaterial in body tissues. To address these issues, pectin can be chemically modified or mixed with other polymers to alter its properties [9,10,11].

The recent growth in hybrid biomaterial research involves a focus on combining biopolymers and inorganic agents. Organosilicon compounds are unique hybrid biomaterials. Porous silicon materials are known to undergo degradation in stomach acid, and silicone functions effectively as a material appropriate for drug encapsulation, transmission, and release [12,13]. Silicon-based biomaterials adhere cells to their surface [14,15,16,17] and are recognized for their tissue adhesion properties during skin wound sealing [17] and adhesion to tooth tissues [18]. Polysaccharide–silica hybrid materials have been investigated as nanocomposite adsorbents [19,20] and biomaterials [21,22,23], and for tissue engineering applications [24]. Silica can be employed to increase the functional qualities of pectin gels, particularly their adhesion.

We previously investigated how the replacement of calcium ions with iron ions [25] and the addition of carrageenan [26] and chitosan [27] alter the biocompatibility and tissue adhesion of pectin hydrogels. Each of the approaches described above involved altering the structure of the pectin gel network. As demonstrated in this study, forming a pectin–silica gel network could represent a promising approach to enhancing mechanical and adhesive properties. A number of studies have been published on pectins and silica hybrid gels. In particular, pectin–silicate gel materials were used to obtain a delivery system for probiotics and medicinal substances [28,29,30,31]. In addition, pectin was used to improve the properties of nano-dentine cement based on tri- and di-calcium silicate [32]. However, to alter the pectin–gel network with the incorporation of silica, an experimental approach differing from that used in most previous studies is required. In previous studies [33,34], the authors often used pre-prepared SiO_2_ (nano)particles for gelation with pectins. In particular, tetraethoxysilane (TEOS) was often used as a source of silica, which was subsequently mixed with pectin [35]. In other experiments, calcium pectinate beads were produced initially before being coated with silica [36]. In the present study, we examined a system formed by mixing pectin and TEOS before gelling. During gelling, the acid hydrolysis of TEOS to -O-Si(OH)_2_-O- can enable the establishment of a covalent bond with pectin’s second or tertiary hydroxyl groups [37]. The interaction between pectin and silica is expected to be stronger under these conditions compared to treating pre-prepared SiO_2_ material with pectin. The functional properties of this hydrogel system, which are important for tissue engineering, have not been previously studied. The incorporation of covalent crosslinking into the pectin–TEOS system is predicted to improve gel mechanical properties, serosa adhesion, and biocompatibility.

In this study, we aim to investigate pectin–TEOS gels, focusing on their mechanical properties, serosal adhesion, and biocompatibility.

## 2. Results and Discussion

### 2.1. Characterization of Pectin–TEOS Hydrogels

The amount of TEOS in the pectin–TEOS hydrogels did not affect their density; however, it decreased their water content and increased their pH (Table 1). The low pH of the pectin–TEOS gel is dependent on the acidity of the pectin and silica gel; notably, pectin is an acidic polysaccharide. The hardness, elasticity, and Young’s modulus of the AP-T1.50 hydrogel were also higher than those of pectin–TEOS hydrogels with less TEOS content (Table 2).

The results are comparable with those of previous work, which found that the hardness of silica-coated calcium pectinate beads increased with silicon content [38]. With increasing TEOS content during gelation, -Si-O-Si- bond formation was possible; thus, silica gel might form in the pectin–TEOS gel network. It has previously been shown that gel hardness and Young’s modulus can reflect the strength and the number of linkages, respectively [39]. It can be assumed, therefore, that the increased number of bridging structures (-O-Si(OH)_2_-O-) between pectin molecules may also cause increasing hardness and Young’s modulus. However, the mechanism of increased elasticity with the addition of TEOS remains poorly understood. It is possible that the increase in elasticity could also occur due to an increase in the number of weak bonds between polymer chains, such as hydrogen bonds and Van der Waals forces. These bonds are capable of breaking under the influence of deformation force and rapidly recovering, maintaining the elasticity of the gel network.

### 2.2. Swelling Studies

**Figure 1:**
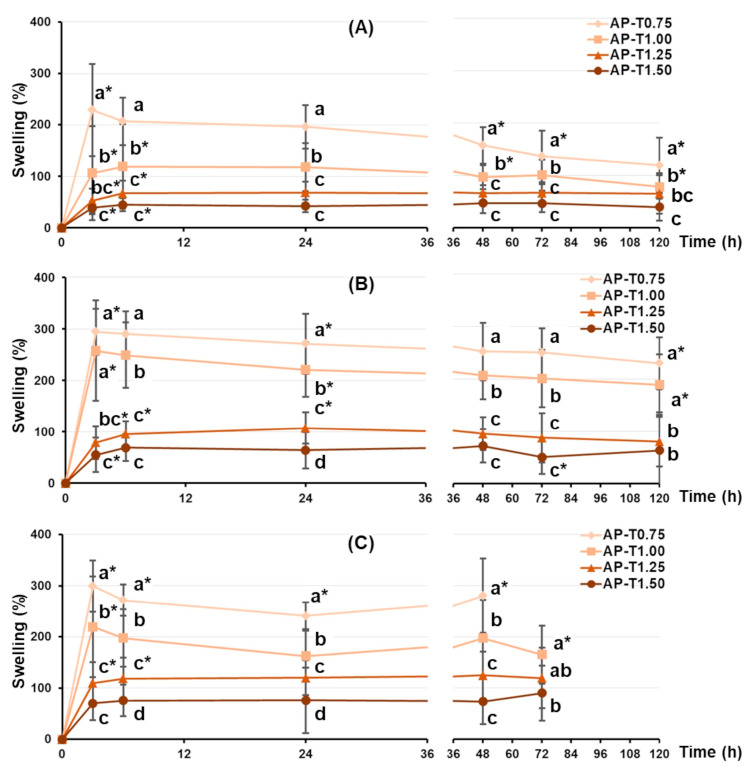
The swelling behavior of the pectin–TEOS hydrogels during 120 h incubation in Hanks’ solution at pH 5.0 (**A**), 7.4 (**B**), and 8.0 (**C**) at a temperature of 37 °C. Different lowercase letters indicate differences (*p* < 0.05) between pectin–TEOS hydrogels. *—*p* < 0.05 compared to the previous incubation time point for the respective gel sample. *n* = 8.

The swelling behavior of pectin–TEOS gels was found to be sensitive to TEOS content and pH. All pectin–TEOS gels gradually swelled for 3 h and then remained at this size for 48 h in Hanks’ solution at pH 5.0, 7.4, and 8.0 (Figure 1). The swelling degrees of AP-T0.75, AP-T1.00, AP-T1.25, and AP-T1.50 were 228, 106, 53, and 39%, respectively, in Hanks’ solution at pH 5.0 (Figure 1A). At pH 7.4, AP-T0.75, AP-T1.00, AP-T1.25, and AP-T1.50 swelled to levels of 294, 257, 79, and 55%, respectively (Figure 1B). The weight of all pectin–TEOS gels gradually declined after 48 h in Hanks’ solution at pH 5.0 and 7.4. The swelling degrees of AP-T0.75, AP-T1.00, AP-T1.25, and AP-T1.50 were 300, 220, 110, and 70%, respectively, in Hanks’ solution at pH 8.0 (Figure 1C). AP-T0.75 was dissolved after 48 h in Hanks’ solution at pH 8.0. AP-T1.00, AP-T1.25, and AP-T1.50 were dissolved after 72 h in Hanks’ solution at pH 8.0.

The swelling of the dried pectin–TEOS gels incubated for 120 h in Hanks’ solution was investigated. Considering the potential use of pectin hydrogels in tissue engineering, the pH level of the incubation medium was determined by the acidity of the extracellular fluid (pH 7.4). It is recognized that tissue and cell damage during implantation procedures can decrease pH to 5.0 during acute inflammation or cause elevation to 8.0 during chronic inflammation [40,41,42]. Therefore, the effect of TEOS on the swelling behavior of the pectin hydrogel was investigated at pH 5.0, 7.4, and 8.0. Comparing hydrogels that reached equilibrium after 120 h of incubation revealed that the pectin–TEOS hydrogel’s capacity to swell at pH 5.0, 7.4, and 8.0 decreased with increasing TEOS content. This finding suggests that the pectin–TEOS material would be more stable during implantation and would have a smaller surface area available for protein adsorption than pectin gel.

### 2.3. Serosal Adhesion of Pectin–TEOS Hydrogels

**Figure 2:**
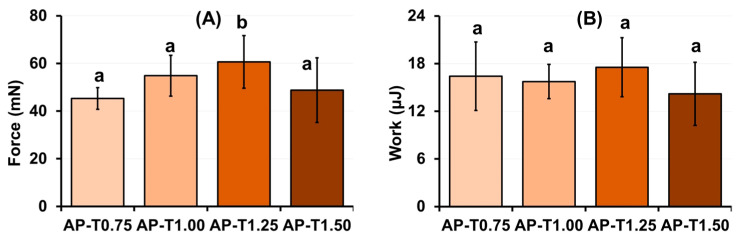
The strength of adhesion (**A**) and work of adhesion (**B**) of pectin–TEOS hydrogels. Different lowercase letters indicate differences (*p* < 0.05) between pectin–TEOS hydrogels. *n* = 8.

The serosal adhesivity of the pectin–TEOS hydrogels was investigated (Figure 2). The adhesion strength of the pectin–TEOS hydrogel AP-T1.25 was 34 and 11% higher than that of the pectin–TEOS hydrogels AP-T0.75 and AP-T1.00 that contained a lower amount of TEOS (Figure 2A). However, the adhesion strength of the pectin–TEOS hydrogel AP-T1.50, containing more TEOS compared to the serosa, did not differ from that of AP-T0.75 and AP-T1.00. The work of adhesion of the pectin–TEOS hydrogel AP-T1.25 did not differ from that of the pectin–TEOS hydrogels AP-T0.75, AP-T1.00, and AP-T1.50 despite the higher adhesion strength (Figure 2B).

In this study, we did not clarify how pectin–TEOS gels adhere to the serosa. Adhesion between adhesives and tissues occurs through intermolecular forces. These forces include covalent bonds, hydrogen bonds, ionic and electrostatic interactions, and van der Waals forces [43]. The structure of mesopolysaccharides, which form the low-adhesion serosal surface covering visceral organs, is not well understood [44]. The adhesion to the serosa, likely mucoadhesion, might involve two stages [45,46]. During the wetting phase, the contact area between the adhesive surface and the serosa initially expanded. Interpolymer contact then resulted in adherence [47]. During an adhesion test, the intertwined chains, which created physical links and weak chemical interactions, stopped the gel from separating from the serosa. The polymer chains forming the gel network might not readily interact with the serosal substrate. The galacturonan backbone likely did not aid the pectin gel in adhering to the serosa. Pectin’s neutral side chains did not join in gel crosslinking; therefore, they might possibly interpenetrate with serosal mesopolysaccharides.

Apple pectin AU202, with a degree of methyl esterification of −82%, contains a low proportion of free carboxyl groups. Films of highly methyl-esterified pectin have been found to adhere to the lung surface more strongly compared to low-esterified pectin [8,48,49]. Electrostatic repulsion influenced the adhesion of blended hydrogels to serous membranes. Silica-based biomaterials are known for their tissue adhesion properties during skin wound sealing [17] and adhesion to tooth tissues [18]. The silica surface has been demonstrated to produce a net negative charge, mostly due to deprotonation of the silanol group, and may most likely absorb cations via electrostatic forces at pH > 2 [50]. The drop in AP-TEOS gel adhesion is not well understood. It may possibly result from a reaction between pectin and TEOS during the formation of polymers. During the preparation of pectin–TEOS gels, TEOS reacted with galacturonic acid residues, linking them to the second and third hydroxyl groups [37]. Thus, pectin hydroxyl groups could not form hydrogen bonds on the serosa surface.

### 2.4. Protein Adsorption on the Pectin–TEOS Hydrogels

Biomaterial implantation during surgery alters tissue homeostasis, resulting in inflammation marked by protein exudation and phagocyte recruitment [51]. Serum protein adsorption is one of the first processes to occur when an implanted biomaterial interacts with body fluids [52]. Serum protein adsorption initiates a sequence of tissue reactions, including complement system activation and leukocyte attachment, known as the foreign-body response (FBR) [53]. The numerous proteins that make up blood serum are a complicated system with a wide range of sizes, concentrations, biological functions, and chemical activities that affect how they adsorb and how quickly they are transported to the surface [54]. Small proteins with low surface affinity and larger concentrations, such as albumin, are included in the reversible initial adsorption. Larger proteins with high surface affinity may gradually displace irreversible adsorption caused by numerous cooperative protein–surface contacts, protein–protein intermolecular interactions, or protein unfolding. Hanks’ solutions containing fetal bovine serum (FBS) or bovine serum albumin (BSA) were therefore utilized to estimate irreversible and reversible protein adsorption by pectin–TEOS gels.

FBR decreases the survival rate of bioengineered materials by creating a scar-like fibrous capsule that isolates the implant. It is evident that a more promising pectin–silicate gel composition is one that is characterized by lower protein adsorption while maintaining tissue adhesion capabilities. Understanding the pH at which adsorption occurs, in addition to the kinetics of protein adsorption, is critical for assessing the risk of developing FBR based on the TEOS content of the hydrogel.

**Figure 3:**
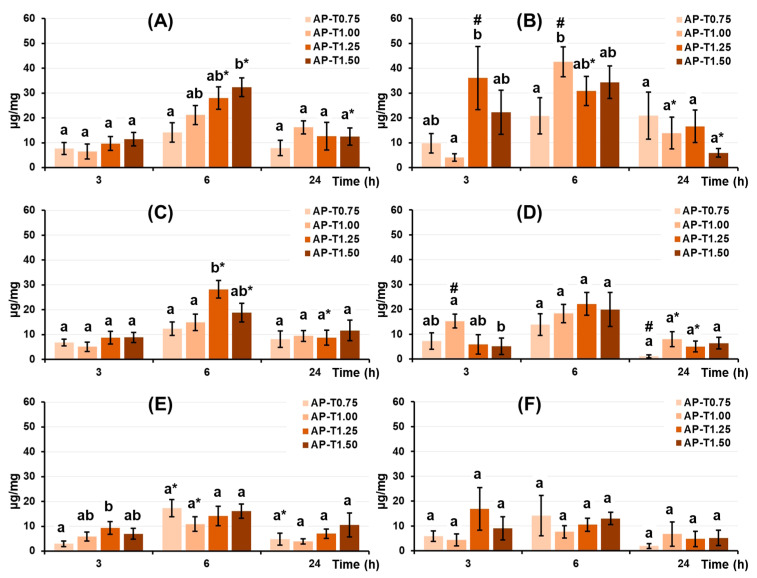
FBS protein adsorption (left column) and BSA adsorption (right column) expressed per unit surface area by pectin–TEOS hydrogels during incubation in Hanks’ solution at pH 5.0 (**A**,**B**), 7.4 (**C**,**D**), and 8.0 (**E**,**F**) at 37 °C. Different lowercase letters indicate differences (*p* < 0.05) between pectin–TEOS hydrogels. *—*p* < 0.05 compared to the previous time point. #—*p* < 0.05 compared between serum proteins and BSA. *n* = 8.

The dried pectin–TEOS gel samples were incubated in Hanks’ solution containing 10% FBS or BSA 0.2 mg/mL at 37 °C to examine their protein-adsorption capacity. Adsorption of serum proteins by pectin–TEOS hydrogels was found to be pH-dependent. Maximum adsorption of fetal bovine serum FBS proteins (Figure 3A,C) by pectin–TEOS gels was observed after 6 h of incubation and decreased with further incubation at pH 5.0 and 7.4. Maximum adsorption of BSA (Figure 3B,D) by pectin–TEOS gels was observed after 3 h of incubation and decreased with further incubation at pH 5.0. The adsorption of FBS proteins (Figure 3E) and BSA (Figure 3F) by pectin–TEOS gels did not alter during 24 h of incubation at pH 8.0 and 7.4. The pectin–TEOS gels AP-T1.25 and AP-T1.50 absorbed 28 and 32 μg/mg of FBS proteins and 31 and 34 μg/mg of BSA, respectively, at pH 5.0. The pectin–TEOS gels AP-T0.75 and AP-T1.00 absorbed 14 and 21 μg/mg of FBS proteins and 21 and 46 μg/mg of BSA, respectively, at pH 5.0.

Blood serum is a complex system of many proteins, such as albumin, fibrinogen, immunoglobulins, and vitronectin, with albumin being the most abundant protein found therein [55]. In studying serum protein adsorption on pectin–TEOS gels, it is therefore important to consider the properties of BSA. Hanks’ solutions containing FBS or BSA were utilized to study serum protein adsorption by pectin–TEOS gels. Electrostatic interactions could drive protein adsorption to pectin gel surfaces, given the presence of dissociated carboxyl groups. Apple pectin AU202 with a degree of methyl esterification of −82% was used in our study; thus, there was a low proportion of free carboxyl groups that could be charged. Moreover, the gel samples contain an identical concentration of AU202 adsorbed with different amounts of proteins. Silica has a high affinity for proteins [19], and silica gel’s structure features a non-crystalline network of silicon atoms to form siloxane bonds (Si-O-Si). It has been shown that pectin–TEOS gels consist of -C-O-Si- and -Si-O-Si- bonds and -Si-OH groups [37]. Proteins interact with silica through a combination of ionic and hydrophobic interactions [56]. The molecular features of the protein determine whether ionic or hydrophilic/hydrophobic forces are of greater importance [56]. Salt addition alters the ionic interactions, which changes how proteins bind and thus their retention time [56]. The unreacted surface hydroxyl groups remaining after polymerization have been shown to exist in dissociated form (Si-O-) and can draw in any positively charged particle at pH > 4 [56]. The reason for the significant increase in protein adsorption by pectin–TEOS with higher TEOS content in Hanks’ solution at pH 7.4 remains unclear and may be due to pectin–silica interaction in the gel network.

The comparison of BSA and FBS adsorption revealed that BSA adsorption was higher than that of FBS after 3 h at both pH 5.0 and 7.4, in addition to after 6 h at pH 5.0. The obtained data confirm the Vroman effect, which describes the competition of different proteins in the FBS for adsorption on the surface of the material [54]. This competitive phenomenon seemingly does not occur in relation to the BSA solution, allowing more protein molecules to be adsorbed. Concurrently, no variations in the protein adsorption of BSA and FBS were seen at pH 8.0, most likely due to BSA’s low adsorption at this pH.

### 2.5. Characterization of Biocompatibility of Pectin–TEOS Hydrogels

FBR, which leads to poor implant integration with native tissue, is the main issue encountered in biomaterials such as hydrogels. An FBR to biomaterials is viewed as a sequence of events: initial protein adsorption and complement activation, followed by activation of leukocytes and macrophages [53]. In this study, we assessed how pectin–TEOS gels impact hemolysis, complement, and macrophage activity in vitro to understand their role during the early stages of FBR.

#### 2.5.1. Hemolysis Assay

The level of hemolysis was determined to assess the hemocompatibility of pectin–TEOS gels. The level of hemolysis induced by the incubation of whole human blood with pectin–TEOS gels is shown in Table 3. The coefficient of induced hemolysis did not exceed 5% when human blood was incubated with pectin–TEOS hydrogels. The pectin–TEOS gels at a concentration of 2–5 mg/mL induced a hemolysis ratio ranging from 0.5 to 4.5%. The pectin–TEOS AP-T0.75, AP-T1.00, and AP-T1.25 at a concentration of 10 mg/mL induced hemolysis ratios of 3.2, 2.4, and 2.6%, respectively. The pectin–TEOS AP-T1.50 at a concentration of 10 mg/mL exceeded 5% and induced a hemolysis ratio of 6.3%.

Pectin–TEOS gels demonstrated acceptable hemocompatibility. A high level of hemolysis suggests that biomaterials are incompatible with erythrocytes; thus, biomaterials should exhibit a hemolysis ratio under 5% (Standard Practice for Assessment of Hemolytic Properties of Materials, ASTM F756, 2017 [57]). Pectin-based biomaterials have demonstrated good hemocompatibility [58,59,60]. These results correlate with previously obtained data [61,62]. Silicon-based materials do not lead to hemolysis when they circulate in the blood [63,64,65]. Thus, the combination of pectin and silicon provides good hemocompatibility. Another event important to biocompatibility is complement activation.

#### 2.5.2. Complement Activation

**Figure 4:**
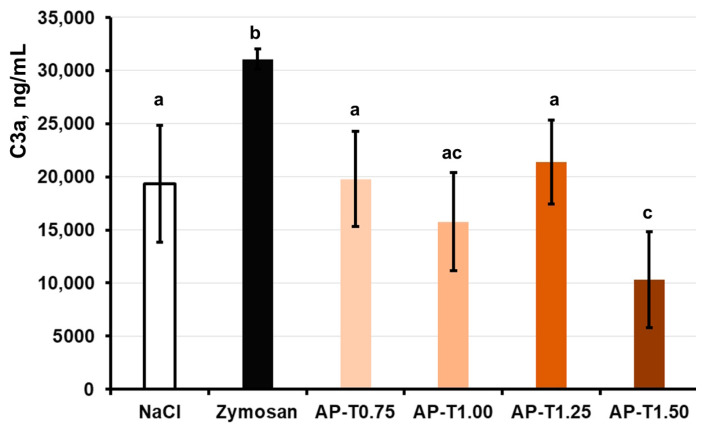
Effect of AP-T0.75, AP-T1.00, AP-T1.25, and AP-T1.50 hydrogels on C3a production in the whole blood in vitro. Different lowercase letters indicate differences (*p* < 0.05) between pectin–TEOS hydrogels. *n* = 8.

The release of the C3a complement component in human blood failed to change after co-incubation with pectin–TEOS gels AP-T0.75, AP-T1.00, and AP-T1.25 and was similar to that of the negative control (NaCl) samples (Figure 4). Blood samples incubated with pectin–TEOS hydrogel AP-T1.50 exhibited a C3a concentration two times lower compared to the negative control (NaCl).

In general, the lack of complement cascade stimulation indicated that the pectin–TEOS gels were biocompatible. The complement system involves many serum proteins that, when triggered, aid in spotting foreign material and starting an innate immune reaction. When serum C3 protein comes into contact with a foreign surface and forms an active C3a fragment, this indicates that the alternative pathway has activated the complement system [66]. In this study, the production of C3a was measured in human blood incubated with the pectin gel in vitro to establish the capacity of the hydrogels to activate the alternative complement cascade. The pectin–TEOS gels, excluding AP-T1.50, did not promote the release of C3a compared to the saline samples (negative control). The levels of C3a were reduced for all pectin–TEOS gels compared to zymosan-treated blood samples (positive control). Zymosan A at a final dose of 0.100 mg/mL was employed as a positive control because it is known to exhaust the alternate pathway of complement activation [67]. The nonspecific adsorption of complement proteins and the accumulation of C3b have been proposed as possible initiators of complement activation by biomaterial surfaces [54]. It is recognized that pectins directly interact with the C3 complement component [61,62,68]. The pectin–TEOS gels contain an identical concentration of AU202 and different silica content. The AP-T1.50 (maximum silica content) gel decreased the concentration of C3a in the blood after incubation. The lowest C3a level in the incubation medium with AP-T1.50 could be due to pectin–TEOS gel AP-T1.50’s strong protein adsorption capacity. Complement system proteins, including C3a protein, can be adsorbed on the gel surface, reducing their concentration in the medium. Data on the influence of silica on complement activation remain inconclusive. Blood collection devices for medical complement activation analysis that contain silica microparticles for clotting whole blood are available [69]. The silica nanoparticles must be coated with proteins to mitigate complement activation [70]. Silica particles are responsible for the complement system activation that leads to some forms of pulmonary inflammation [71]. Nevertheless, in our study, pectin–TEOS gels did not activate or inhibit activation of the complement system. Serum protein adsorption and complement activation are followed by leukocyte adhesion on the surface of the biomaterial.

#### 2.5.3. Peritoneal Macrophage Adhesion on the Pectin–TEOS Hydrogels

**Figure 5:**
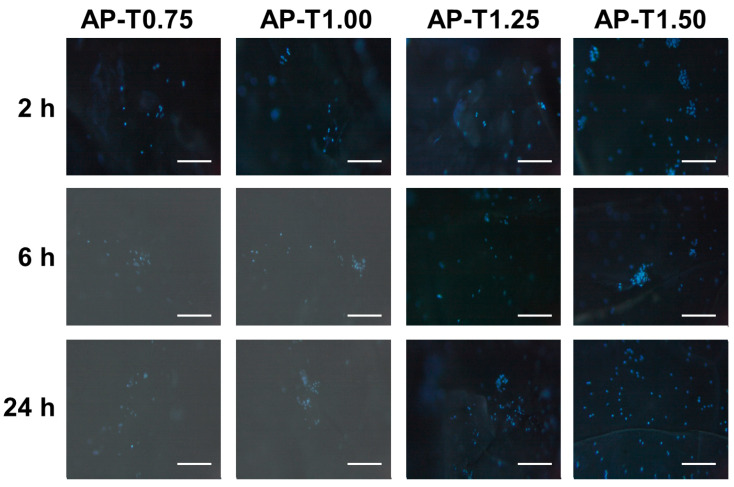
Peritoneal leukocyte adhesion to the surface of AP-T0.75, AP-T1.00, AP-T1.25, and AP-T1.50 hydrogels. Cells were stained with DAPI. Bar—100 µm.

The pectin–TEOS gels were incubated with a suspension of mouse peritoneal macrophages in Hanks’ solution. The peritoneal macrophages were found to adhere to the pectin–TEOS hydrogels (Table 4). The number of leukocytes that adhered to the AP-T0.75 hydrogel increased with incubation time. The number of cells attached to the AP-T0.75 hydrogel was 2.6 and 2.3 times greater after 6 and 24 h than after 2 h, respectively. The cell adhesion on the AP-T1.00, AP-T1.25, and AP-T1.50 hydrogels did not change over time. The maximum adhesion of leukocytes on AP-T1.50 was 5.9 and 2.1 times higher compared to AP-T0.75 after 2 and 24 h of incubation. Representative images of the hydrogel surface with adherent cells are shown in Figure 5.

Protein adsorption on implanted biomaterials impacts later adhesion and the activation of polymorphonuclear leukocytes and macrophages [52]. Based on the findings of a number of studies, cell adhesion is promoted by non-specific protein adsorption [72]. Contrariwise, the so-called “protein coat” hides the biomaterial made from immune cells [73]. Albumin, the most abundant protein in human blood, can inhibit the adherence of inflammatory cells, reducing biomaterial-induced inflammatory responses [74,75]. In our previous study, we reported that molecules on the surface of pectin-based hydrogel cells can adhere in the presence or absence of the so-called “protein coat” [25,26,27,76]. In the present study, the maximum level of serum protein adsorption was noted after 6 h of incubation; in comparison, maximum leukocyte adhesion to three out of four pectin–TEOS gels was noted after 2 h of incubation in Hanks’ solution (Figure 3 and Table 4). Silicon-based biomaterials adhered cells to their surface [13,14,15,16]. In our study, an increase in the content of silica in pectin–TEOS gel led to an increase in leukocyte adhesion.

## 3. Conclusions

Hybrid pectin–TEOS gels were prepared in this study. The silica content in the pectin–TEOS gels influences the mechanical properties (hardness, Young’s modulus, and elasticity), degree of swelling, and serosal adhesion. A gel consisting of 1.5 M TEOS (AP-T1.50) was found to have the highest hardness, Young’s modulus, and elasticity and the lowest degree of swelling. A gel consisting of 1.25 M TEOS (AP-T1.25) was found to have the highest strength of adhesion to serosa. AP-T1.50 adsorbed serum proteins and adhered leukocytes at pH 7.4 to a greater extent than other gels. Thus, serosal adhesion combined with high mechanical stability and interaction of the pectin–TEOS gel with proteins and cells, which determine interactions with body tissues after implantation, represents the advantages of the pectin–TEOS gel for the development of tissue adhesives and biomaterials. In the future, the benefits of hybrid gels of polysaccharide–inorganic material and the pectin–TEOS gels examined in this work will be crucial for creating novel biomaterials or enhancing current biomaterials with adhesive qualities. Further research is required to determine the characteristics of pectin–TEOS gels when they are implanted in lab animals.

## 4. Materials and Methods

### 4.1. Materials

Apple pectin AU202 (galacturonic acid—78.0%, degree of methyl esterification—82%, Mw—506 kDa) (Herbstreith & Fox GmbH, Nuremberg, Germany) and tetraethoxysilane (TEOS) (Fluka, Paris, France) were used in this study. Hanks’ balanced salts solution powder (PanEco, Moscow, Russia) containing NaCl 140 mM, KCl 5 mM, CaCl_2_ 1 mM, MgSO_4_ 0.4 mM, MgCl_2_ 0.5 mM, Na_2_HPO_4_ 0.3 mM, KH_2_PO_4_ 0.4 mM, D-glucose 6 mM, and NaHCO_3_ 4 mM was used to obtain Hanks’ solution. Zymosan A (Sigma-Aldrich, St. Louis, MO, USA) was used as a positive control in the complement activation evaluation.

### 4.2. Preparation of Pectin–TEOS Gels

**Figure 6:**
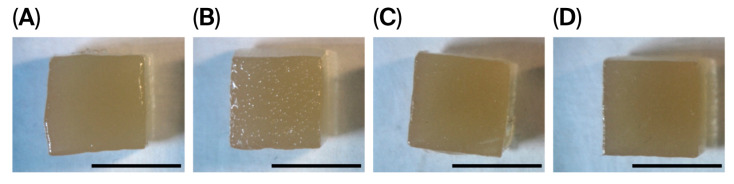
Representative images of hydrogel cubes of AP-T0.75 (**A**), AP-T1.00 (**B**), AP-T1.25 (**C**), and AP-T1.50 (**D**) hydrogels. Bar—10 mm.

**Figure 7:**
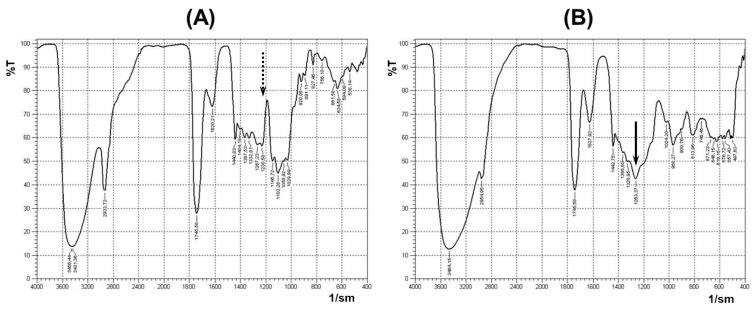
FTIR spectra of pectin AU202 (**A**) and pectin–TEOS gel AP-T0.75 (**B**). The dashed arrow shows the absence of the absorption band; the solid arrow shows the presence of the absorption band.

The sol–gel technique was used to prepare the pectin–TEOS gels, using a method described previously [37]. The pectin–TEOS hydrogels contained 0.75, 1.00, 1.25, and 1.50 M TEOS, were produced, and were labeled AP-T0.75, AP-T1.00, AP-T1.25, and AP-T1.50, respectively. To produce the above hydrogels, a mixture of 6.65% AP solution with 8.55, 11.4, 14.25, or 17.0 mL of TEOS was prepared, diluted to 57.8 mL with distilled water, and heated to 90 °C with continuous magnetic stirring (200 rpm) for 40 min to improve mixing. The resulting mixtures were placed on a Petri dish (d = 90 mm), and HCl (0.06 mL) was added drop by drop on the surface. The reaction mixtures were incubated for 24 h. The hydrogel samples (10 mm × 10 mm × 10 mm) were washed thrice with 70% ethanol solution. The final compositions of the four pectin–TEOS gel samples are shown in Table 5. The appearance of the pectin–TEOS hydrogels is shown in Figure 6. FTIR spectra of pectin AU202 and pectin–TEOS hydrogel AP-T0.75 are shown in Figure 7.

The sol–gel technique was used to prepare pectin–TEOS gels as described previously [37]. Previously, absorption peaks in FTIR spectra at 1246 cm^−1^ and 802 cm^−1^ indicated the production of C-O-Si bonds in the produced pectin–silica gels [37]. The acquired data support the observation of a significant absorption band at 1440–1237 cm^−1^ of the FTIR spectrum during the substitution of hydroxyl groups of polysaccharide and the creation of covalent bonds. Synytsya et al. (2003) found that absorption bands at 1250 cm^−1^ indicate the production of C-O-C bonds after the partial acetylation of pectin [77]. Absorption bands at 1230–1270 cm^−1^ and 800–850 cm^−1^ were seen after the production of C-O-S/P bonds in sulfated or phosphate polysaccharides [78]. Thus, an evaluable difference between pectin and pectin-SiO_2_ systems was revealed using FTIR spectroscopy. The obtained data may indicate the presence of covalent bonds in pectin–TEOS gels. However, FTIR spectroscopy data may not be sufficient to prove a covalent bond between pectin and silica. Unfortunately, the solid-phase ^13^C HMR method was not used in the present study, which is a limitation of the study.

### 4.3. Characterization of Pectin–TEOS Hydrogels

The pH was determined for hydrogel aqueous homogenates (1:10 (*w*/*v*)) using an S20 SevenEasy™ pH meter (Mettler-Toledo AG, Schwerzenbach, Switzerland). The weight of 1 cm square hydrogel cubes (*n* = 8) was measured (AG245, Mettler Toledo International, Greifensee, Switzerland) to determine the density as weight/volume. The water content was calculated as described previously [25].

### 4.4. Texture Characterization

A puncture test (probe P/2, diameter 2 mm, depth 6 mm) for the AP-T0.75, AP-T1.00, AP-T1.25, and AP-T1.50 hydrogel cubes (10 mm × 10 mm × 10 mm) was performed using a TA-XT Plus (Texture Technologies Corp., Stable Micro Systems, Godalming, UK) instrument at room temperature [27]. Hardness, Young’s modulus, and elasticity were calculated using Exponent Stable MicroSystems (Version V6.1.4.0, Godalming, UK).

### 4.5. Swelling Characterization of Pectin–TEOS Gels

During this stage, 4 mg of dry pectin–TEOS hydrogel cubes was incubated in Hanks’ solution at pH 5.0, 7.4 and 8.0 supplemented with 25 mM HEPES for 3, 6, and 24 h at 37 °C. The swelling degree (SD) was determined as previously described [27]. The swelling degree (SD) was determined using Equation (1):

SD% = ((S1 − S0)/S0) × 100,(1)

where S0 and S1 are the initial weight and weight after a determined incubation time.

### 4.6. Tissue Adhesion Assay

The gel adhesion to the serosa of the rat small intestine was measured to evaluate the bioadhesive properties of the gels. The adhesion tests were performed using a texture analyzer, TA-XT Plus (Texture Technologies Corp., Stable Micro Systems, Godalming, UK). A gel probe was prepared as described previously [27]. The probe compressed the rat small intestine serosa at 50 mN compression force for 20 s. The force of probe separation from the tissue after 20 s of pressing was recorded and calculated using Exponent Stable MicroSystems (Version V6.1.4.0, Godalming, UK) [27].

### 4.7. Protein Adsorption by Pectin–TEOS Hydrogels

Dried gel samples were incubated in Hanks’ solution (as in the swelling experiments) containing 10% of FBS or BSA 0.2 mg/mL as described previously [27]. Sample aliquots were collected from wells after 3, 6, and 24 h of incubation and centrifuged at 1000× *g* for 20 min at 4 °C, followed by measurement of the protein concentration in the supernatant using the Micro BCA Protein Assay Kit (Thermo Scientific™, Waltham, MA, USA). The amount of adsorbed protein was adjusted to hydrogel weight.

### 4.8. Biocompatibility of the Pectin–TEOS Hydrogels

#### 4.8.1. Hemolysis Ratio Determination

The hemolysis ratio was measured after incubation of whole blood with pectin gel beads. The dried gel beads at different concentrations (2, 4, and 8 mg/mL) were placed in sterile 2 mL microcentrifuge tubes (Eppendorf, Leipzig, Germany), and 0.3 mL of blood was added to each tube and incubated for 1 h at 37 °C without stirring, as described previously [76].

#### 4.8.2. Complement Activation Evaluation

Venous blood was collected from healthy volunteers and placed in vacuum tubes (Improvacuter, Guangzhou Improve Medical Instruments, Guangzhou, China) after obtaining written informed consent. The protocol was approved by the Ethical Committee of the Komi Science Centre of the Russian Academy of Sciences. Complement activation evaluation was performed by measuring C3a levels, as previously described [27,79]. Zymosan A at a final concentration of 0.100 mg/mL was used as a positive control and pyrogen-free 0.9% saline (NaCl, 0.05 mL) as a negative control.

#### 4.8.3. Peritoneal Macrophage Adhesion Evaluation

Peritoneal leukocytes were obtained from mice, as previously described [80]. Peritoneal macrophage adhesion evaluation was performed as described previously [27]. In brief, after pectin–TEOS hydrogel incubation in cell suspension (2 × 10^6^ cells/mL), the gel material was treated with 4′,6-diamidino2-phenylindole (DAPI) (Sigma-Aldrich, St. Louis, MO, USA). The animal study was approved by the Ethical Committee of the Komi Science Center of the Russian Academy of Sciences (no. 2022-1003, date of approval: 10 March 2022).

### 4.9. Statistical Analysis

Data are presented as means ± standard deviations (*n* = 8). One-way ANOVA with Tukey’s honest significance test was applied to determine statistically significant differences in independent measurements. ANOVA for repeated measurements was used to determine statistically significant differences between time points. Values of *p* ≤ 0.05 were considered statistically significant.

## Figures and Tables

**Table 1:** Effect of TEOS on the properties of pectin hydrogels.

Hydrogel	Density (g/cm^3^)	Water Content (%)	pH
AP-T0.75	0.97 ± 0.13 ^a^	93.8 ± 0.2 ^a^	3.34 ± 0.07 ^a^
AP-T1.00	0.93 ± 0.02 ^a^	93.1 ± 0.2 ^b^	3.46 ± 0.09 ^ab^
AP-T1.25	0.90 ± 0.03 ^a^	92.2 ± 0.4 ^c^	3.50 ± 0.04 ^b^
AP-T1.50	0.94 ± 0.03 ^a^	92.4 ± 0.4 ^c^	3.45 ± 0.10 ^ab^

Different lowercase letters indicate differences (*p* < 0.05) between pectin–TEOS hydrogels. *n* = 8.

**Table 2:** Mechanical properties of pectin–TEOS hydrogels.

Hydrogel	Hardness (kPa)	Young’s Modulus (kPa)	Elasticity (mm)
AP-T0.75	340 ± 20 ^a^	1237 ± 141 ^a^	1.77 ± 0.09 ^ab^
AP-T1.00	325 ± 42 ^a^	1183 ± 101 ^a^	1.72 ± 0.12 ^a^
AP-T1.25	406 ± 29 ^a^	1189 ± 63 ^a^	1.82 ± 0.07 ^ab^
AP-T1.50	631 ± 190 ^b^	1588 ± 229 ^b^	1.95 ± 0.28 ^b^

Different lowercase letters indicate differences (*p* < 0.05) between pectin–TEOS hydrogels. *n* = 8.

**Table 3:** The effect of pectin–TEOS hydrogels on hemolysis ratio (%) in vitro.

Hydrogel	Hydrogel Concentration in Blood		
	2 mg/mL	5 mg/mL	10 mg/mL
Distilled Water (Positive control)	100	100	100
0.9% NaCl (Negative control)	0	0	0
AP-T0.75	0.47 ± 0.29 ^a^	1.58 ± 0.61 ^a^	3.24 ± 1.02 ^a^
AP-T1.00	1.60 ± 0.48 ^a^	1.11 ± 0.38 ^a^	2.43 ± 0.34 ^a^
AP-T1.25	0.65 ± 0.22 ^a^	3.40 ± 1.59 ^a^	2.65 ± 0.50 ^a^
AP-T1.50	1.32 ± 0.68 ^a^	4.55 ± 2.63 ^a^	6.30 ± 1.33 ^b^

The hemolysis induction coefficient is expressed as a percentage. Different lowercase letters indicate differences (*p* < 0.05) between pectin–TEOS hydrogels. *n* = 8.

**Table 4:** The number of peritoneal leukocytes adhered to the surface (cells/mm^2^) of pectin–TEOS hydrogels.

Hydrogel	Incubation Time		
	2 h	6 h	24 h
AP-T0.75	104 ± 41 ^a^	271 ± 124 ^a^ *	237 ± 171 ^a^
AP-T1.00	349 ± 166 ^ac^	369 ± 206 ^a^	244 ± 192 ^a^
AP-T1.25	479 ± 264 ^bc^	422 ± 155 ^a^	491 ± 86 ^b^
AP-T1.50	613 ± 184 ^b^	482 ± 222 ^a^	507 ± 174 ^b^

Different lowercase letters show difference (*p* < 0.05) between hydrogels. *—*p* < 0.05 compared to the previous time point. *n* = 8.

**Table 5:** Final compositions of pectin–TEOS hydrogels.

Gel	AU202 (%)	TEOS (M)
AP-T0.75	4	0.75
AP-T1.00	4	1.00
AP-T1.25	4	1.25
AP-T1.50	4	1.50

## Data Availability

The data that support the findings of this study are available from the corresponding author upon reasonable request.
